# Association Between Neutrophil–Lymphocyte Ratio and Frailty: The Chinese Longitudinal Healthy Longevity Survey

**DOI:** 10.3389/fmed.2021.783077

**Published:** 2022-01-03

**Authors:** Weihao Xu, Yuanfeng Liang, Zhanyi Lin

**Affiliations:** ^1^Department of Cardiology, Guangdong Provincial Cardiovascular Institute, Guangdong Provincial People's Hospital, Guangdong Academy of Medical Sciences, Guangzhou, China; ^2^Department of Geriatrics, Guangdong Provincial Geriatrics Institute, Guangdong Provincial People's Hospital, Guangdong Academy of Medical Sciences, Guangzhou, China

**Keywords:** inflammatory marker, neutrophil-lymphocyte ratio, frailty, frailty index, old age

## Abstract

**Background:** Inflammation has been reported to play an important role in frailty syndrome. The neutrophil–lymphocyte ratio (NLR) has recently emerged as an informative marker for systematic inflammation. However, few studies have examined the association between NLR and frailty. This study aims to examine the association between NLR and frailty in community-dwelling older adults.

**Methods:** Community-dwelling older adults aged ≥ 65 years in the 2011 (*n* = 2,354) and 2014 (*n* = 2,458) waves of the Chinese Longitudinal Healthy Longevity Survey (CLHLS) were included. Frailty status was determined using the 38-item frailty index (FI) and categorized into “robust” (FI ≤ 0.1), “pre-frail” (0.1 < FI ≤ 0.21), or “frail” (FI > 0.21). NLR was calculated using a derived formula: NLR = (white blood cell–lymphocyte)/lymphocyte.

**Results:** A total of 3,267 participants were finally included. In cross-sectional analyses, participants with higher NLR levels had increased likelihood of frailty [the 3^rd^ quartile: adjusted odds ratio (OR) = 1.29; 95% confidence interval (CI): 1.02–1.63; the 4^th^ quartile: OR = 1.59; 95% CI: 1.23–2.02) compared with those in the 1^st^ quartile group. During the 3-year follow-up, 164 of the 1,206 participants, robust or pre-frail at baseline, developed frailty, and 197 of the 562 participants, robust at baseline, developed pre-frailty or frailty. Among the robust and pre-frail participants in 2011, after multivariate adjustment, those in the 4^th^ quartile group had a higher frailty incidence than those in the 1^st^ quartile group (OR = 2.06; 95% CI: 1.18–3.59). Among the robust participants in 2011, those in the 4^th^ quartile group also had a higher pre-frailty or frailty incidence than those in the 1^st^ quartile group (OR = 1.95; 95% CI: 1.07–3.55).

**Conclusion:** Among community-dwelling older adults, higher NLR levels were found to be associated with increased odds of prevalent and incident frailty.

## Introduction

Frailty is a common geriatric syndrome characterized by age-associated morphological and physiological changes across multiple systems and organs, resulting in progressive decline of physiological reserve, reduced resilience, and increased vulnerability when exposed to stressors (1). Frail older adults have a significantly increased risk of various adverse outcomes, including falls, cardiovascular disease, hospitalization, nursing home admission, poor surgical outcomes, disability, and death (2–8). Data from epidemiological studies show that the prevalence of frailty is high among community-dwelling older adults: approximately 6, 12, and 25% of persons aged 65–74, 75–84, and 85+ years, respectively (9). Frailty is an emerging health and economic burden for not only individuals and families but also the society.

Understanding the pathophysiological mechanisms of frailty is of great importance for developing prevention and intervention strategies. Collective evidence suggests that chronic inflammation plays an important role in frailty occurrence (10). Previous studies have suggested that higher levels of inflammatory markers are associated with loss of muscle mass and function (lower strength and lower-extremity performance) (11, 12). Furthermore, inflammatory markers are also correlated with accelerated loss of mobility and physical activity (13). In addition, a meta-analysis also showed that frailty and pre-frailty are associated with increased levels of inflammatory markers, particularly CRP and IL-6 (14). The neutrophil–lymphocyte ratio (NLR) has recently emerged as an informative marker for systematic inflammation and has been linked to poor prognosis in several diseases, such as cancers (15), chronic obstructive pulmonary disease (COPD) (16), acute coronary syndrome (ACS) (17), and acute cerebral hemorrhage (ICH) (18). However, few studies have examined NLR as an inflammatory marker of frailty (19, 20). Hou et al. found that a higher NLR level (3^rd^ and 4^th^ quartile of NLR of study sample) is associated with frailty in elderly patients with coronary heart disease (CHD) (19). A study by Nishijima and colleagues also showed an association between frailty and NLR in older adults with cancer (20). However, all previous studies focused on the association between NLR and frailty were performed in disease-specific (CHD or cancer) study populations and with small sample sizes, so it is unclear whether the association persists in community-dwelling older adults.

In the present study, we analyzed data from the Chinese Longitudinal Healthy Longevity Survey (CLHLS) to examine the association between NLR and frailty among community-dwelling older adults in China.

## Methods

### Study Design and Population

The CLHLS is a nationwide study of community-dwelling older adults aged ≥ 80 years. Face-to-face interviews were used to collect extensive information, including socio-demographic characteristics, psychological characteristics, and physical and cognitive health conditions. This study began enrolling participants in 1998 from 22 of China's 31 provinces. Follow-up interviews were conducted every 2 years before the third wave (2000 and 2002) and then every 3 years after the third wave (2005, 2008, 2011, and 2014). The study added new samples (adults aged ≥ 80 years in the second wave and adults aged ≥ 65 years in the third and subsequent waves) to replenish participants who had died or were lost to follow-up. In 2011 and 2014, an ancillary study was conducted in eight longevity areas: Laizhou City, Xiayi County, Zhongxiang City, Mayang County, Yongfu County, Sanshui District, Chengmai County, and Rudong County. In this ancillary study, a blood test was added and blood sample was collected voluntarily from study participants with informed consent. Further details about the recruitment strategy and study design of CLHLS have been described elsewhere (21). The current analyses used data from the 2011 and 2014 waves of the CLHLS. In the 2011 wave, 9,679 participants aged 65–114 years were interviewed. Among them, 2,354 participants received blood tests and 2,160 had NLR data. In the 2014 wave, 7,107 participants aged 65–117 years were interviewed. Among them, 2,458 participants received blood tests and 2,401 had NLR data.

### Frailty Index

The outcome of interest in this study is frailty, as determined by the frailty index (FI). We constructed the FI following a standard procedure (22). The FI counts health deficits. Health deficits could be defined as symptoms, signs, disabilities, and diseases (18). Health deficits included in the FI must fulfill the following criteria: (1) be associated with health status, (2) show an increase with age and be higher than 1%, (3) not saturate at an early age, and (4) cover several physiological systems. Each health deficit was scored as 0 (absence), 1 (presence), or missing. For each participant, the FI score was calculated by summing the deficits present and dividing by the number of deficits included, and it ranged from 0 to 1. We constructed a 38-item FI (Supplementary Table S1) following an established research using data from CLHLS (23). In our study, participants who had ≥ 30 items were included. After the FI was calculated, all participants were categorized as robust (FI ≤ 0.1), pre-frail (0.1 < FI ≤ 0.21), or frail (FI > 0.21) (24).

### Covariates

Covariate variables in this study included age, sex, marital status (married and living with spouse, divorced, widowed, or never married), years of education, current smoking status, current drinking status, current exercise status (Assessed by the question: “Do you exercise regularly now?” Exercise refers to purposeful physical activity, such as walking, playing ball, running, qigong, etc.), and body mass index (BMI).

### Neutrophil–Lymphocyte Ratio

Fasting blood samples were collected by trained nurses and tested in a local hospital or a local office of the China Center for Disease Prevention and Control. Blood routine tests only included white blood cell (WBC) count (10^9^/L) and lymphocyte count (10^9^/L). Thus, the NLR used in this study is a derived NLR (dNLR), calculated as: (WBC – lymphocyte)/lymphocyte (25). NLR levels were categorized by two ways: (1) quartiles and (2) dichotomous groups (“normal” [<3] vs. “high” [≥ 3] inflammation) (26).

### Statistical Analysis

To maximize the sample size, all participants had NLR data in the 2011 wave, and participants who were replenished in the 2014 wave and also had NLR data were combined and used in cross-sectional analyses.

Baseline characteristics of the combined sample were reported and compared according to the different frailty statuses (robust, pre-frail, frail). Due to skewed distribution, continuous variables (age, BMI, NLR, and FI) are described as median and interquartile range (IQR) and were compared by the Kruskal–Wallis test. Categorical variables are described as frequencies and were compared by a chi-square test. Cross-sectional association between NLR levels and frailty (robust or pre-frailty vs. frailty) was examined using logistic regression analysis. Prospective associations of NLR levels with a follow-up of (1) frailty (robust or pre-frail participants in the 2011 wave) and (2) pre-frailty or frailty (robust participants in the 2011 wave) were also examined using logistic regression analysis. Three models were built for the logistic regression analysis. In addition to a crude model, model 1 was adjusted for age and sex. Model 2 was additionally adjusted for marital status, education years (≥ 5 years), current smoking status, current drinking status, current exercise status, and BMI.

All analyses were performed using the SPSS 26.0 for Windows (SPSS Inc., Chicago, IL). *P* < 0.05 were considered statistically significant.

## Results

### Characteristics of Study Population

**Figure 1:**
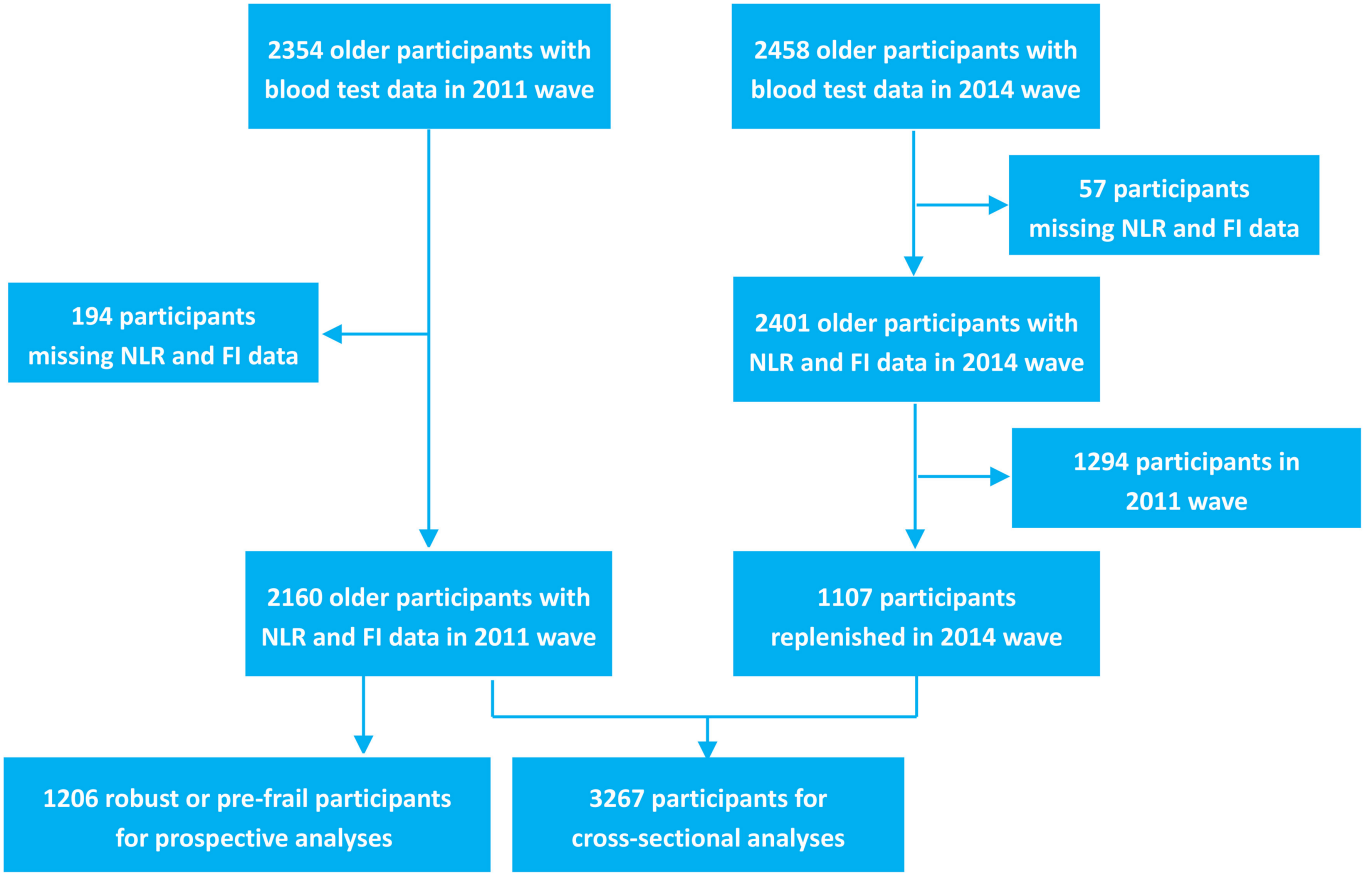
Flowchart of the study identification process.

Figure 1 shows the details of study selection process. Of the 3,267 participants, 843 were robust (25.8%), 1,042 were pre-frail (31.9%), and 1,382 were frail (42.3%). The characteristics of included participants are presented in Table 1. The median age was 87.0 years (IQR, 77.0–98.0 years). Females accounted for 56.4% of the participants. Age, BMI, NLR, proportion of females, marital status, current smoking status, current drinking status, education level, current exercise status and comorbidity [hypertension, diabetes, heart disease and stroke or cerebrovascular disease (CVD)] were significantly different among the three groups.

**Table 1:** Characteristics of the community-dwelling older adults of the CLHLS (*n* = 3,267, according to frailty status).

	**Frailty status**			
	**Robust (***n*** = 843)**	**Pre-frail (***n*** = 1,042)**	**Frail (***n*** = 1,382)**	***P*** **-value**
Age [years, median (IQR)]	77.0 (70.0, 85.0)	85.0 (77.0, 93.0)	95.0 (87.0, 101.0)	<0.001
Gender (female, %)	347 (41.2)	561 (53.8)	933 (67.5)	<0.001
BMI [median (IQR)]	21.9 (19.8, 24.2)	20.9 (18.8, 23.8)	20.1 (17.9, 22.9)	<0.001
Marital status (*n*, %)				<0.001
Married and living with spouse	488 (57.9)	390 (37.4)	250 (22.2)	
Divorced/widowed/never married	355 (42.1)	652 (62.6)	1132 (81.9)	
Current smoker (*n*, %)	206 (24.4)	175 (16.8)	118 (8.5)	<0.001
Current drinker (*n*, %)	189 (22.4)	161 (15.5)	113 (8.2)	<0.001
Exercise at present (*n*, %)	212 (25.1)	172 (16.5)	101 (7.3)	<0.001
Education years (≥ 5 years, %)	320 (38.0)	179 (17.2)	156 (11.3)	<0.001
Comorbidity				
Hypertension	151 (17.9)	323 (31.0)	497 (36.0)	<0.001
Diabetes	13 (1.5)	36 (3.5)	132 (9.6)	<0.001
Heart disease	28 (3.3)	79 (7.6)	242 (17.5)	<0.001
Stroke or CVD	12 (1.4)	56 (5.4)	229 (16.6)	<0.001
NLR [median (IQR)]	2.2 (1.7, 2.9)	2.1 (1.6, 2.9)	2.3 (1.7, 3.2)	<0.001
Frailty index scores [median (IQR)]	0.06 (0.03, 0.08)	0.15 (0.12, 0.18)	0.31 (0.24, 0.43)	<0.001

*IQR, interquartile range; BMI, body mass index; CVD, cerebrovascular disease; NLR, neutrophil lymphocyte ratio*.

### Cross-Sectional Association Between NLR Levels and Frailty

In the multivariable adjusted model, participants in the highest two quartiles of NLR groups had significantly greater odds of frailty than those in the lowest quartile (OR = 1.29, 95% CI: 1.02–1.63 for the 3^rd^ quartile, and OR = 1.59, 95% CI: 1.23–2.02 for the 4^th^ quartile). There was a significant trend of increasing cumulative odds of frailty with higher NLR level (*P* for trend < 0.001). When NLR was categorized into dichotomous groups, participants in “high” inflammation state (NLR ≥ 3) had a 46% higher likelihood of frailty (OR = 1.46, 95% CI: 1.21–1.78) than those in normal inflammation state (NLR < 3) (Table 2).

**Table 2:** Cross-sectional association of NLR with frailty in community-dwelling older adults of the CLHLS (*n* = 3,267).

	**Crude model**	**Model 1^a^**	**Model 2^b^**
NLR group 1			
Quartile 1	1.00		1.00
Quartile 2	0.95 (0.78–1.16)	1.07 (0.85–1.34)	0.97 (0.76–1.23)
Quartile 3	1.14 (0.94–1.38)	1.34 (1.08–1.67)**	1.29 (1.02–1.63) *
Quartile 4	1.56 (1.28–1.90)***	1.77 (1.41–2.22)***	1.59 (1.23–2.02)***
*P*-value for trend	<0.001	<0.001	<0.001
NLR group 2			
Normal (<3)	1.00	1.00	1.00
Abnormal (≥3)	1.53 (1.31–1.79)***	1.58 (1.32–1.89)***	1.46 (1.21–1.78)***

*NLR, neutrophil lymphocyte ratio*.

a *Adjusted for age and sex*.

b *Adjusted for age, sex, marital status, education years (≥5 years), current smoking status, current drinking status, current exercise status and body mass index*.

* *P < 0.05*,

** *P < 0.01*,

*** *P < 0.001*.

### Prospective Association Between NLR Levels and Frailty

During the follow-up period, 164 of the 1,206 robust or pre-frail participants developed frailty. After full adjustment for confounders, participants in the 4^th^ quartile NLR group were associated with a more than 2-fold greater likelihood of incidence of frailty than participants in the 1^st^ quartile group. When NLR was categorized into dichotomous groups, participants in “high” inflammation state (NLR ≥ 3) had an 84% higher likelihood of incidence of frailty than those in normal inflammation state (NLR <3) (Table 3).

**Table 3:** Prospective association of NLR with incidence of frailty in robust or pre-frail (*n* = 1,206) community-dwelling older adults of the CLHLS study cohort.

	**Crude model**	**Model 1^a^**	**Model 2^b^**
NLR group 1			
Quartile 1	1.00	1.00	1.00
Quartile 2	1.28 (0.78–2.11)	1.39 (0.80–2.41)	1.31 (0.75–2.28)
Quartile 3	1.33 (0.80–2.21)	1.61 (0.92–2.82)	1.53 (0.87–2.70)
Quartile 4	1.56 (0.96–2.55)	2.09 (1.21–3.62)**	2.06 (1.18–3.59)*
*P*-value for trend	0.082	0.008	0.009
NLR group 2			
Normal (<3)	1.00	1.00	1.00
Abnormal (≥3)	1.39 (0.93–2.08)	1.65 (1.05–2.60)*	1.84 (1.14–2.97)*

*NLR, neutrophil lymphocyte ratio*.

a *Adjusted for age and sex*.

b *Adjusted for age, sex, marital status, education years (≥5 years), current smoking status, current drinking status, current exercise status and body mass index*.

* *P < 0.05*,

** *P < 0.01*.

During the follow-up period, 197 of the 562 robust participants developed pre-frailty or frailty. After multivariable adjustment, participants in the 4^th^ quartile NLR group had nearly double the risk of incident pre-frailty or frailty that those in the 1^st^ quartile group. When NLR was categorized into dichotomous groups, participants whose NLR ≥ 3 at baseline had more than 2-fold greater likelihood of incident pre-frailty or frailty than those had NLR <3 (Table 4).

**Table 4:** Prospective relationship between NLR and incidence of pre-frailty or frailty in robust (*n* = 562) community-dwelling older adults of the CLHLS.

	**Crude model**	**Model 1^a^**	**Model 2^b^**
NLR group 1			
Quartile 1	1.00		1.00
Quartile 2	0.96 (0.56–1.65)	0.92 (0.52–1.63)	0.93 (0.52–1.65)
Quartile 3	1.18 (0.68–2.07)	1.24 (0.69–2.22)	1.20 (0.66–2.17)
Quartile 4	1.86 (1.07–3.24)*	1.99 (1.10–3.60)*	1.95 (1.07–3.55)*
*P*-value for trend	0.016	0.009	0.014
NLR group 2			
Normal (<3)	1.00	1.00	1.00
Abnormal (≥ 3)	1.92 (1.19–3.09)**	2.01 (1.21–3.33)**	2.11 (1.24–3.59)**

*NLR, neutrophil lymphocyte ratio*.

a *Adjusted for age and sex*.

b *Adjusted for age, sex, marital status, education years (≥5 years), current smoking status, current drinking status, current exercise status and body mass index*.

* *P < 0.05*,

** *P < 0.01*.

## Discussion

In this large prospective cohort study of more than 3,000 community-dwelling older adults, we found that a higher NLR level was associated with increased odds of prevalent and incident frailty.

Based on our literature search, few studies have investigated the association between NLR and frailty. Nishijima and colleagues performed a cross-sectional study of 133 elderly patients with cancer to examine the association between NLR and frailty. They used the 36-item Carolina Frailty Index to assess frailty status and reported that patients in the highest tertile NLR group had nearly 4-fold increased odds of frailty (20). Another cross-sectional study by Hou et al. was conducted on 345 elderly patients with coronary heart disease to explore the relationship between NLR and frailty. This study used the Fried frailty phenotype to define frailty and showed that participants in the 3^rd^ and 4^th^ quartile NLR groups had higher odds for frailty (OR = 2.53 and 2.89, respectively) (19). Both these studies had limitations, including cross-sectional design, small sample size, and disease-specific study populations, which limited the generalization of study findings, but they indicated a possible association between NLR and frailty. Our study, based on a large sample of community-dwelling older adults, further demonstrates the association between NLR and frailty by both cross-sectional and prospective analyses.

Although limited studies have focused on the NLR and frailty, there is much evidence exploring the association between NLR and sarcopenia, which is a common geriatric syndrome and is significantly associated with frailty. A recent study by Abete et al. enrolled 1,535 overweight/obese participants and found that NLR is negatively associated with sarcopenic index, as assessed using dual-energy X-ray absorptiometry scanning (27). Studies conducted on patients with cancer have shown that increasing NLR is associated with sarcopenia in a dose-response manner (26). Considering that sarcopenia is a strong risk factor for frailty, the above evidence might indirectly indicate the association between NLR and frailty.

Inflammation has been widely considered to be associated with frailty. Most frail older adults have chronic inflammation, especially those with comorbid sarcopenia (28, 29). Previous research has found that inflammation is associated with decreased synthesis and activity of insulin-like growth factor I (IGF1), which plays an essential role in muscle regeneration and maintenance of muscle integrity (30). There is also evidence showing that IGF1-mediated anabolism can be inhibited by IL-1α, IL-6, and that both TNF inhibitors *in vitro* and IL-6 can reduce the production of IGF1 and IGF-binding protein 3 (31). In addition, observational studies have reported that high IL-6 levels and low IGF1 levels synergistically correlate with lower muscle strength and power, which is the main clinical manifestation of frailty (32). Furthermore, studies have shown that NLR is significantly correlated with IL-6, CRP, and TNF-α (33–35). However, the mechanism underlying the association between NLR and frailty is unclear. We speculate that a higher level of NLR is secondary to an existing high systematic inflammation level, and NLR might serve as an indirect marker of inflammation. This hypothesis needs to be verified in further studies.

To the best of our knowledge, our study, for the first time, examined the prospective association between NLR and frailty. Our results point toward the possible clinical application of NLR since the NLR data can be easily available from a routine blood test. More attention should be paid to community-dwelling older adults or elderly patients with higher NLR level to take early preventive measures to reduce the occurrence of frailty as well as the adverse outcomes associated with frailty. It should be noted that the optimal cut-off point of NLR is not yet established. Although we found the cut-off value of ≥ 3 for NLR, which was recommended by a previous study (26), was able to predict future pre-frailty and frailty, more longitudinal studies are still warranted to further explore the best cut-off value for NLR in older adults. Strengths or our study also include a large sample size and nationally representative study population. We also acknowledge several limitations of this study. First, we could only use the FI to define the frailty status of participants in the CLHLS cohort study. Considering that the association between NLR and frailty might be involved in the mechanism of frailty, the Fried frailty phenotype should be the ideal assessment tool for frailty under this circumstance. Future studies using the Fried frailty phenotype to define frailty are warranted to further investigate the association between NLR and frailty. Second, the analytic sample for the prospective associations between NLR and frailty was relatively small due to follow-up loss and death. Third, our study was conducted among community-dwelling older adults, and the study results may not generalize to elderly patients, especially those with inflammatory diseases. Finally, residual confounding factors (polypharmacy, sarcopenia, depression, fruit/vegetable/protein consumption, etc.) for frailty may still be present, although we adjusted for several confounders.

## Conclusions and Implications

In summary, the present study confirms the association between NLR and frailty in community-dwelling older adults. The NLR relies on information obtained from a simple blood test, which is available in epidemiological studies; can be administered in various care and clinical settings; and serves as a risk factor for frailty.

## Data Availability Statement

The original contributions presented in the study are included in the article/Supplementary Material, further inquiries can be directed to the corresponding author.

## Ethics Statement

The studies involving human participants were reviewed and approved by Peking University and Duke University. The patients/participants provided their written informed consent to participate in this study.

## Author Contributions

WX wrote and participated in all aspects of this research, including the field investigation. YL analyzed the data and interpret the results. ZL designed this research. All authors reviewed the manuscript and agree to be accountable for the content of the work.

## Funding

This study was supported by National Key R&D Program of China (2018YFC2000301).

## Conflict of Interest

The authors declare that the research was conducted in the absence of any commercial or financial relationships that could be construed as a potential conflict of interest.

## Publisher's Note

All claims expressed in this article are solely those of the authors and do not necessarily represent those of their affiliated organizations, or those of the publisher, the editors and the reviewers. Any product that may be evaluated in this article, or claim that may be made by its manufacturer, is not guaranteed or endorsed by the publisher.
