# Supplementary material for: Association Between Neutrophil–Lymphocyte Ratio and Frailty: The Chinese Longitudinal Healthy Longevity Survey
Source: Front Med (Lausanne). 2022 Jan 3;8:783077. doi: 10.3389/fmed.2021.783077 (PMC8761893; doi:10.3389/fmed.2021.783077)
Supplement: Supplementary file 1 [file Table_1.DOCX]

**Association between** **Neutrophil–lymphocyte Ratio and Frailty: The Chinese Longitudinal Healthy Survey**

Weihao Xu^1,2†^, Yuanfeng Liang^2†^, Zhanyi Lin^1,2*^

**Table S1. Variables Used to Construct the Frailty Index**

| Variables | | Data Type | Cut-off point |
| --- | --- | --- | --- |
| 1 | Self-reported health | Ordinal | V. good=0, good=0.25; so so=0.5, bad=0.75, very bad=1 |
| 2 | Feel fearful or anxious | Ordinal | Always=1, often=0.75, sometimes=0.5, seldom=0.25, rarely or never=0 |
| 3 | Feel useless with age | Ordinal | Always=1, often=0.75, sometimes=0.5, seldom=0.25, rarely or never=0 |
| 4 | Bathing | Ordinal | Without assistance=0, one part assistance=0.5, more than one part assistance=1 |
| 5 | Dressing | Ordinal | Without assistance=0, one part assistance=0.5, more than one part assistance=1 |
| 6 | Toileting | Ordinal | Without assistance=0, one part assistance=0.5, more than one part assistance=1 |
| 7 | Transferring | Ordinal | Without assistance=0, one part assistance=0.5, more than one part assistance=1 |
| 8 | Continence | Ordinal | Without assistance=0, one part assistance=0.5, more than one part assistance=1 |
| 9 | Feeding | Ordinal | Without assistance=0, one part assistance=0.5, more than one part assistance=1 |
| 10 | Visual function | Ordinal | Can see and distinguish=0, can see only=0.5, can’t see=1, blind=1 |
| 11 | Rhythm of heart | Binary | >=80bpm=1; <80bpm=0 |
| 12 | Hand behind neck | Ordinal | Both hands=0, left hand=0.5, right hand=0.5, neither hand=1 |
| 13 | Hand behind lower back | Ordinal | Both hands=0, left hand=0.5, right hand=0.5, neither hand=1 |
| 14 | Able to stand up from sitting | Ordinal | Yes, without using hands=0, Yes, using hands=0.5, no=1 |
| 15 | Able to pick up a book from the floor | Ordinal | Yes, standing=0, Yes, sitting=0.5, no=1 |
| 16 | Number of times suffering from serious illness in the past two years | Ordinal | Yes=2, no=0 |
| 17 | Hypertension | Binary | Yes=1, no=0 |
| 18 | Diabetes | Binary | Yes=1, no=0 |
| 19 | Heart disease | Binary | Yes=1, no=0 |
| 20 | Stroke or CVD | Binary | Yes=1, no=0 |
| 21 | Bronchitis, emphysema, pneumonia, asthma | Binary | Yes=1, no=0 |
| 22 | Tuberculosis | Binary | Yes=1, no=0 |
| 23 | Cancer | Binary | Yes=1, no=0 |
| 24 | Gastric or duodenal ulcer | Binary | Yes=1, no=0 |
| 25 | Parkinson | Binary | Yes=1, no=0 |
| 26 | Bedsore | Binary | Yes=1, no=0 |
| 27 | Able to hear | Binary | Yes=1, no=0 |
| 28 | Interviewer rated health | Ordinal | Surprisingly healthy=0, relatively healthy=0, moderately ill=0.5, very ill=1 |
| 29 | Look on the bright side of things | Ordinal | Always=0, often=0.25, sometimes=0.5, seldom=0.75, rarely or never=1 |
| 30 | Keep my belongings neat and clean | Ordinal | Always=0, often=0.25, sometimes=0.5, seldom=0.75, rarely or never=1 |
| 31 | Make own decisions | Ordinal | Always=0, often=0.25, sometimes=0.5, seldom=0.75, rarely or never=1 |
| 32 | Housework at present | Ordinal | Almost everyday=0, not daily, but once for a week=0.25, not weekly, but at least once for a month=0.5, not monthly, but sometimes=0.75, never=1 |
| 33 | Able to use chopsticks to eat | Ordinal | Yes=1, no=0 |
| 34 | Number of steps used to turn around a 360 degree turn without help | Interval | >=6 steps=1, <6 steps=0 |
| 35 | Cataract | Binary | Yes=1, no=0 |
| 36 | Glaucoma | Binary | Yes=1, no=0 |
| 37 | Other chronic disease | Categorical | Yes=1, no=0 |
| 38 | Prostate Tumor | Binary | Yes=1, no=0 |
